# Hospital and Institutional News

**Published:** 1920-11-06

**Authors:** 


### HOSPITAL AND INSTITUTIONAL NEWS.

### SIR NAPIER BURNETT'S BIBLICAL ILLUSTRATION.

Continuing his campaign, Sir Napier Burnett recently gave an address at the Primitive Methodist Chapel at Harrow, and frightened the congregation by the suggestion that they might be in for two shillings in the pound, extra to the present rates, in respect of municipal hospital work. The speaker based his remarks on the parable of the Good Samaritan, and said that the doctrine of cowardly subterfuge brought to light in the story was one of the oldest in history. It was adopted by many people to-day in their attitude to hospitals: they were content to pass on the other side and say it was none of their business. Possibly the fault lay in defective dissemination of information about hospitals, and the mass of the population did not appreciate what was being done for the cure of disease, the investigation of causes of disease, and the training of doctors and nurses. A great obligation rested upon the public, because the hospitals supplied them with the doctors and nurses when they were ill that they would not be able to procure if there were no hospitals. The hospitals of the Metropolitan boroughs last year cost for maintenance just over £2,000,000, and the rateable value was about £45,000,000, which meant that for maintenance alone this would represent a rate of about one shilling in the pound. Allowing for what were now voluntary services, the rate would be 2s. It would pay employers to deal with this matter while the hospitals were still voluntary.

### THE BRITISH HOSPITALS' ASSOCIATION.

It will be remembered that the British Hospitals' Association at their annual meeting appointed a small committee to confer with the College of Nursing as to the proper hospital and nursing attitude that should be assumed in respect of the Hours of Employment Bill. As a consequence, we understand, the Executive Council of the British Hospitals' Association are in agreement with the College of Nursing that an average fifty-six-hour week spread over four weeks, with a maximum of ten hours in any one period of twenty-four hours, is a fair proposition to be recommended to the constituent hospitals in this respect. The activities of the British Hospitals' Association are greater than appear to be generally supposed, and another matter under consideration is the Unemployment Act as it affects hospitals. It is hoped that an announcement will shortly be made in respect of how hospitals stand in regard to the Act. A Regional Committee for the East Midlands District was formed at a meeting at the Nottingham General Hospitals on October 21, when Mr. Wade Deacon attended and addressed the meeting, which, largely owing to the tact and skill of the organiser, Mr. W. Banks, Secretary of the Derbyshire Royal Infirmary, was very successful.

### THE GOLDEN SQUARE THROAT HOSPITAL.

The Throat Hospital in Golden Square a few days ago offered its buildings for sale by auction, but we understand that the reserve was not reached. It is proposed to move to Streatham, where the hospital committee has the offer of a site. As is the case with all the special hospitals, the patients come from all parts of London, and therefore, while Streatham would be a convenient locality for many, it would be less convenient for many more, and, in view of the heavy cost of travelling, would put hospital treatment by this particular institution out of reach of not a few. The medical staff feel so strongly on the necessity of a central position for the hospital that they are protesting against the move. It is impossible that the doctors cannot have in their minds as well the inconvenience that would result to themselves, and, under the present system of an unpaid staff, it is quite fair that their views should be duly considered by the hospital committee. It is also urged that the cost of rebuilding and adapting the institution at Streatham would be greater than the cost of equipping the present hospital with the latest improvements, which the medical staff consider is all that is required to make it an up-to-date institution.

### THE "BART.'S" APPEAL.

It would be interesting to have statistics of the success achieved by hospital appeals since the end of the war. We therefore welcome the statement issued by the treasurer of St. Bartholomew's Hospital, who records that the sum of £132,000 has been subscribed to the Peace Year Commemoration Appeal which was issued a year ago. This has enabled the Committee to pay to the bankers the debt due to them a year ago, and has left a balance toward the expenses which will be incurred before the end of the year. Lord Sandhurst reminds the public that the hospital now costs about £160,000 a year to run, so that more than the sum-total raised by the recent appeal is needed annually. While, therefore, peace year is over, the appeal for funds must still be made. The assured income of the hospital has not been adequately increased. That is the serious fact. It is stated that "practically every section of the community throughout the country" responded to the appeal. Since the London hospitals do not seem able to increase the numbers of their annual subscribers in London, will the attempt be made to convert the scattered units which respond to their appeals into regular contributors? For the latter class must be found somewhere.

### HOSPITALS AND WATER SUPPLY.

In a report on water charges the Metropolitan Water Board mentions that a section of the Charges Act regulates the supply to special classes of premises—namely, workhouses, public institutions, etc.—and frees the Board from the obligation to supply these otherwise than by measure. The reason for this is that the consumption of water in such premises may often be altogether out of proportion to the rateable value. In the Bill as originally deposited hospitals were also included in the list, but, as the result of a determined effort on the part of the hospital authorities, the Water Board was precluded from compelling them to take their supplies by meter. The result of this is that many hospitals consume a large quantity of water and pay on the basis of a nominal rateable value. Moreover, the fittings in use frequently contravene the Board's regulations. The Board holds the view that, in order that undue consumption of water may be reduced, the hospitals should be compelled to take their supplies by meter, but that, having regard to the charitable objects for which they are established, there should be a very substantial rebate from the meter charge.

### HEALTH MINISTRY AND POOR-LAW INFIRMARIES.

The newly appointed medical men on the staff of the Ministry of Health are now busily engaged in making a survey of the accommodation and equipment of Poor-law institutions. London infirmaries and sick wards of the Poor-law institutions are being examined and particulars noted of the equipment and staff available for surgical work, also the accommodation for medical cases and maternity. It is thought that when the survey is completed a scheme will be evolved for the classification of cases, as far as possible, so that certain infirmaries will deal mainly with patients requiring surgical treatment, whilst others will deal with medical cases. In the London area many Poor-law institutions have a number of aged inmates who could quite well be accommodated in a suburban district, and the buildings so vacated could with some adaptation be used as ancillaries to the hospitals. Lewisham Board of Guardians has already gone to work on these lines, and thus freed a large number of beds for patients requiring hospital treatment

### A WARDLESS HOSPITAL.

The newest type of hospital appears to be under course of construction in New York. It is to be known as Fifth Avenue Hospital and its scheme is luxurious in the extreme. Built in the form of an X, it will be entirely guiltless of the "old-time" ward and every one of its 300 rooms will be private, open alike to poor and rich. Certainly the scheme has advantages, more especially to the more nervous and sensitive patients, but we wonder if there will not be many a gregarious patient who will wish himself less secluded! The medical men on the staff will occupy the entire first floor, and each apartment will have a private door in order that outside patients may visit their doctor without coming through the hospital. We shall await the completion of the building with interest.

### THE APPEAL CAMPAIGN AT CANTERBURY.

In furtherance of the extensive appeals being made on behalf of the Kent and Canterbury Hospital, a public meeting was held recently in the Chapter House, an ancient place of meeting attached to the cathedral. The Archbishop of Canterbury made a strong appeal on behalf of the hospital, urging its importance in the exercise of the Christian principle of service to one's fellow-men. The meeting was presided over by the Mayor of Canterbury (Councillor James), and other speakers included Lord Northbourne, President of the Hospital, and the venerable Dean of Canterbury, Dr. Wace, who, although in his eighty-fourth year, still takes an active part in the administration of the institution, as Chairman of the Hospital Committee. The speeches will be epitomised and circulated throughout East Kent in a serious effort to increase the number of subscribers. At the same time, an elaborate scheme for a donation of a penny a week from all householders in Canterbury and the surrounding districts will be put into operation. Another feature of the campaign was the Drumhead Service held in the open air on a recent Sunday afternoon, when the Dean officiated at Divine Service, assisted by ministers of various denominations. A combined choir sang the hymns, and the music was supplied by the bands of the East Kent Regiment ("The Buffs") and the Salvation Army. As the result of this picturesque and unique function the hospital benefited to the extent of £82.

### CRITICS AT CARDIFF.

The tense atmosphere of the coal strike would naturally be felt in South Wales, and we noticed a certain amount of grumbling at the recent meeting of the board of King Edward VII. Hospital. One member declared that the trade unionists in the city had lately expressed some hostility because of the waiting list, which had grown to 1,200. To this Mr. Leonard Rea, the superintendent, replied that, notwithstanding a monthly deficit of £3,000, the board was going to open the Edward Nicholl Ward, which would add fifty beds to the hospital. Mr. Rea added that the accommodation for the nursing staff was a practical difficulty. He also pointed out that the hospital was receiving only a limited supply of coal, and unless, as he hoped, a solution could be found, it would be criminal to open an additional ward at the moment. Another member reported a movement in the outlying districts for secession, if the difficulty of admitting patients was not reduced. The chairman, Mr. Jabez Jones, urged the need for further subscriptions, and was told that, in the case of some colliers, the stoppages from their wages for doctors and institutions amounted to no less than 7s. 6d. a week each. We can sympathise with both parties. But it is only fair to remember that the hospital is not responsible for the extent of its task. It is only responsible for the extent of its efforts. It has, in fact, done famously in recent years, but though its limit of capacity has not been reached, we wonder if too great demands are not being made upon it. For this reason we have welcomed the movements for local hospitals in South Wales as tending to relieve the tension. The extension scheme, still in process, contemplated a certain number of beds, but not an unlimited number, and every effort is at present required to attain this standard. The last we heard of the Lord Mayor's appeal was that it had reached £120,000. That sum is largely to balance the present accounts, and to confirm the work already undertaken. The hospital has rapidly grown to an institution with between three and four hundred beds. The public might be told before the present appeal ends how many more beds would be required to admit every one of the 1,200 unfortunate waiting patients immediately. We doubt if the critics themselves in the least realise the financial problem represented by this figure.

### THE POSITION AT BIRMINGHAM.

At present the efforts made to rehabilitate the finances of the Birmingham General Hospital have not attained their aim. A response has been made by many firms and their staffs; but it has not been general or sufficient. Mr. C. H. Saunders, chairman of the board, therefore points out that the existing overdraft of £42,000 is being met by the sale of one-quarter of the hospital's securities Another quarter consists of trust funds which cannot be realised. The remaining half, if sold at the same rate, would enable the hospital to continue till the end of 1922. By that time, if the subscriptions and prices remain at their existing level, the hospital will be able to maintain only 200 beds instead of 370. That is a straightforward statement. We hope that it will bring the facts home, and that a general endeavour will be made to save the situation. The increased support is only a fraction of what is required.

### BIRMINGHAM GENERAL HOSPITAL MAGAZINE.

The policy of the Committee of the General Hospital, Birmingham, is to get in touch with the worker and the small giver, and to this end the hospital is bringing out a magazine called *G.H.B.*, the first number of which, price one penny, is before us. It is a bright little paper, not only because of the orange and blue of its cover. The contents are simply written and easily read, and they convey the message that is intended. Naturally, the new paper pushes the Birmingham General Hospital League, which, we understand, is gathering in many helpers in industrial circles. At the General the Committee is advertising for a food supervisor, a title not much used in hospital circles, and meaning, we take it, something like a super-housekeeper. It is hoped that some specially trained woman, who does not merely seek a stepping-stone to a matron's post, will come forward and show what she can do to increase the importance of this branch of hospital work.

### HOUSE-TO-HOUSE COLLECTION.

The Great Northern Hospital has organised a house-to-house collection in the whole area of its operations (roughly seventy square miles), the effort taking place from November 1 to 6 inclusive. Nearly seven hundred voluntary collectors were expected to take part in the scheme. A new form of propaganda at the Great Northern Central Hospital is the magazine *Progress*, which chronicles the wants and the activities of the hospital. This periodical is nicely produced and well illustrated, and judging by the number of advertisements is not a heavy burden on the costs of appeal.

### THE MIGHTY MITE AT KING'S COLLEGE HOSPITAL.

Stirred by sympathy for their fellows, 1,200 members of the Amalgamated Chapels of Messrs. W. H. Smith and Son, of 186 Strand, recently unanimously imposed upon themselves the obligation of each contributing for a period of six months the sum of twopence per week towards the funds of King's College Hospital, in order to help it in its great and increasing work of mercy and healing for the sick and suffering. Two of the members a few days ago visited the hospital and handed the Secretary the sum of £40, representing the members' weekly contribution for a period of one month. This generous and noble determination of the members to assist King's College Hospital at a time of great need, when no less a sum than an additional £60,000 a year must be raised, to enable it to carry on the increastd amount of work which it is called upon to do for those overtaken by accident, sickness, or disease, and who cannot receive the necessary and satisfactory treatment outside, is most gratifying.

### HOSPITAL POUND DAY.

A Pound Day is to be held at the London Homœopathic Hospital, Great Ormond Street and Queen Square, W.C. 1, on Tuesday, November 9, when the Countess of Donoughmore has graciously consented to be present from 11 a.m. until 5 p.m. to receive the gifts. The occasion affords an admirable opportunity for those who are cognisant of the value of the work performed at the hospital to show their sympathy in a most practical form, and her ladyship expresses the hope that the appeal will meet with a worthy response.

### NOTTINGHAM SANATORIUM.

The Health Committee of the Nottingham Corporation have, after strong opposition from various bodies in the locality of the proposed site, succeeded in getting their scheme approved, and steps are to be taken to purchase an estate with a view to erecting a permanent sanatorium for the accommodation of some 200 men, women, and children. For some years prior to the war the need for special treatment for those suffering from tuberculosis was very great, and temporary accommodation had to be provided at an isolation hospital. With the great increase of this disease during and since the war, it seems very evident that adequate accommodation should be provided as soon as possible. Old buildings are ill adapted for conversion into hospitals and little more expense is incurred in building proper blocks, thus promoting efficiency in treatment. The objections raised are familiar enough, and we are sorry to see, as usual, religious bodies among the objectors. But first, experience alone shows that the very people who objected are quick to change when they see the benefits that follow; and secondly, it would be welcome evidence of enlightenment in public opinion if similar mass meetings could be held up and down the country to protest against the bulk of the people living in sordid and unhealthy surroundings and thus becoming predisposed to the disease. We hope the delay in completing buildings (other than luxury buildings), so noticeable all over the country, will be absent when this proposal materialises. The State grant for three-fifths of the cost will be available, since the scheme has been passed by the date fixed; and will lessen the financial burden very considerably.

### LONDON HOSPITAL PATIENTS AT MORDEN HALL.

Mr. G. E. Hatfield, of Morden Hall, near Mitcham, who throughout the war equipped and maintained his residence as a hospital for wounded soldiers, lately offered the place to the London Hospital, which has now installed forty women and children as patients there. Mr. Hatfield, who lives in an improved cottage on the estate, and visits his "guests" regularly, is bearing the whole cost of their maintenance himself. A bachelor, who has always taken a lively interest in hospital work, Mr. Hatfield is the head of Taddy's tobacco factory, which was lately closed in consequence of a strike. There are several people whose houses are too big for them in these times, and if they would follow his example the waiting lists of the London hospitals would be reduced at a moment when the expenses of building and the scarcity of labour prolong the shortage of accommodation, even when the funds exist to pay for further extensions.

### "TIN-FOIL" BEDS.

In connection with the account which a short while since appeared in the columns of The Hospital, describing the endowment of beds by the collection of tinfoil, it is interesting now to learn that another bed—making the eighth—has been recently provided through that means by the Ancient Order of Druids.

### A SUSSEX EXPERIMENT.

The Committee of the Royal West Sussex Hospital, Chichester, has decided on an interesting plan to replace the fall in subscriptions from the well-to-do which till lately formed a large part of the hospital's income. The plan is to appeal to all employed men and women, lads and girls in the neighbourhood for systematic support, and to ask all employers, "including those who employ domestic servants only," to act as the machinery for collection. Weekly donations from a halfpenny to a few pence are wanted, and the hospital will supply collecting-boxes for those employers who have only one or two persons on their staff. Each employer will be asked to hand the appeal to his employees, and, if they express a wish to support it, to make it simple for them to do so. This is an interesting attempt in an old cathedral town in an agricultural county, where, except for that of the retail shops in the city and that of the farmers outside, there is little organisation for the collection of weekly gifts. But if all is decentralised, all is personal, and we hope that personal goodwill, as so often before, will supply the place of a central pay office. The attempt is creditable, and we hope to learn of its success.

### DOCTORS AND MUNICIPAL TREATMENT.

At Barnes Urban District Council a petition signed by fifteen medical practitioners in the district was presented. The object was to protest against the treatment of cases at the Maternity and Child Welfare Centre coming within the scope of the medical officer of health, the contention being that it is "treatment" and not preventive medicine. The petition expressed the opinion that all medical men in the area who care to take it up, at the standard fee per session, should be offered this work. The request was also made that anæsthetics given at the dental clinic to children attending for dental treatment should be offered to local medical practitioners. The clerk was instructed to reply that treatment is not undertaken at the child welfare centre, and that as regards the dental clinic, that work was organised by the Surrey Education Committee, to whom a copy of the petition has been forwarded.

### SIR GEORGE BEATSON.

It has been decided to recognise the services of Sir George Beatson, who retired last May from the post of chairman of the Council and of the Executive Committee of the Scottish Branch of the Red Cross Society, by presenting him with his portrait. The painter is to be Mr. Fiddes Watt. The Society's debt to Sir George Beatson was recorded in a resolution setting forth his unfailing guidance during the war, and to his success in organising the Scottish Branch and the Voluntary Aid Detachments before 1914. His professional qualifications and thorough knowledge of military organisation were referred to; and, indeed, it would be difficult to exaggerate the value of his work. Scotland has now become a central council branch of the parent society, with the same rights in Scotland as the latter has in England. A new constitution, giving the members in each county two annually-elected representatives on the council, has been approved. Lord Blythswood succeeds Sir George Beatson as chairman.

### THIS WEEK'S DRUG MARKET.

Things seem to be going from bad to worse, and buyers are more reluctant than ever. Prices continue to sag and apparently attractive offers do not attract purchasers. A substantial reduction in the price of santonin is expected, and supplies are more plentiful. Makers of morphine and codeine have reduced their quotations slightly; the price of opium is nominally unchanged. Phenacetin, phenazone, aspirin, benzoates, salicylates, resorcin, and in fact most of the synthetic drugs, are extremely quiet, and the price tendency is distinctly easier. Potassium bromide is again cheaper. The small demand for cod-liver oil has resulted in an easing in the price.

